# Identification of Immune-Related Prognostic Genes and LncRNAs Biomarkers Associated With Osteosarcoma Microenvironment

**DOI:** 10.3389/fonc.2020.01109

**Published:** 2020-07-24

**Authors:** Tao Zhang, Yingli Nie, Haifa Xia, Yanbin Zhang, Kailin Cai, Xiangdong Chen, Huili Li, Jiliang Wang

**Affiliations:** ^1^Department of Anesthesiology, Union Hospital, Tongji Medical College, Huazhong University of Science and Technology, Wuhan, China; ^2^Department of Dermatology, Wuhan Children's Hospital (Wuhan Maternal and Child Healthcare Hospital), Tongji Medical College, Huazhong University of Science and Technology, Wuhan, China; ^3^Department of Orthopaedics, Union Hospital, Tongji Medical College, Huazhong University of Science and Technology, Wuhan, China; ^4^Department of Gastrointestinal Surgery, Union Hospital, Tongji Medical College, Huazhong University of Science and Technology, Wuhan, China

**Keywords:** osteosarcoma, immune, prognosis, biomarker, tumor microenvironment

## Abstract

Osteosarcoma (OS) is the most common malignancy of the bone that occurs majorly in young people and adolescents. Although the survival of OS patients markedly improved by complete surgical resection and chemotherapy, the outcome is still poor in patients with recurrent and/or metastasized OS. Thus, identifying prognostic biomarkers that reflect the biological heterogeneity of OS could lead to better interventions for OS patients. Increasing studies have indicated the association between immune-related genes (IRGs) and cancer prognosis. In the present study, based on the data concerning OS obtained from TARGET (Therapeutically Applicable Research to Generate Effective Treatments) database, we constructed a classifier containing 12 immune-related (IR) long non-coding RNAs (lncRNAs) and 3 IRGs for predicting the prognosis of OS by using the least absolute shrinkage and selection operation Cox regression. Besides, based on the risk score calculated by the classifier, the samples were divided into high- and low-risk groups. We further investigated the tumor microenvironment of the OS samples by ESTIMATE and CIBERSORT algorithms between the two groups. Finally, we identified three small molecular drugs with potential therapeutic value for OS patients with high-risk score. Our results suggest that the IRGs and IR-lncRNAs–based classifier could be used as a reliable prognostic predictor for OS survival.

## Introduction

Osteosarcoma (OS) is the most common malignancy of bone that occurs majorly in young people and adolescents (1), which accounts for ~4–5 per 1,000,000 per year (2). Surgical resection combined with chemotherapy has increased long-term survival rates to 60–70% for patients with the localized OS, but only 20–30% for patients with recurrent and/or metastasized OS (3, 4). Besides, the outcome of OS patients may be distinctly different even with the same stage. Thus, identifying prognostic biomarkers that reflect the biological heterogeneity of OS could lead to better interventions for patients.

Much attention has been paid to immune oncology for its impressive clinical benefits in a variety of malignancies. Immune-related genes (IRGs) and immune infiltrating cells have been considered as determining factors for regulating tumor development and progression (5, 6). The breakthrough of immunomodulatory therapies targeting the programmed death 1 (PD-1)/PD-1 ligand (PD-L1) signaling, a pathway crucial for impairing the immune system, has shown considerable success in multiple cancers by promoting antitumor immune function (7). Besides, studies have shown that both PD-1 and PD-L1 were significantly upregulated in OS patients and correlated with the prognosis (8, 9), and a recent study found that blockade of PD-1/PD-L1 signaling dramatically promoted the activity of cytotoxic T lymphocytes, inhibiting the tumor growth and increasing the survival rate in the mouse model of metastatic OS (10). Besides, tumor microenvironment (TME), where the tumor cells are located, is increasingly thought to play vital roles in tumor development and progression (11). Tumor microenvironment consists of extracellular matrix molecules, stromal cells, immune cells, and inflammatory mediators (12). As one of the major non-tumor cellular populations in the TME, infiltrating immune cells have been shown highly associated with responses to treatments and clinical outcomes of cancers. Tumor with high immune infiltration was associated with a better prognosis (13–16). Besides, bone has a highly specialized immune environment, and multiple immune signaling pathways play important roles in bone homeostasis (3). These evidences suggest that the application of immune-based prognostic biomarkers in OS is a potential. Furthermore, based on this, we can explore the underlying mechanisms and identify potential therapeutic drugs, so as to bring new insights into the improvement of the prognosis of OS patients.

Recent evidence suggests that long non-coding RNAs (lncRNAs) play important roles in regulating the development and activation of multiple immune cells through controlling the dynamic transcriptional programs (17) and involve carcinogenesis and metastasis (18). Immune-related (IR) lncRNAs have shown to act as biomarkers for predicting the risk of cancer patients of gliomas (19) and glioblastoma multiforme (20).

Several IRG-based signatures have been constructed to predict the risk of patients with different cancer types, such as lung cancer (21), glioblastoma (22), gastric cancer (23), and renal papillary cell carcinoma (24). As for OS, the prognostic values of IRGs and IR-lncRNA have still not been explored. In the present study, in an effort to assess the potential utility of IRGs and IR-lncRNAs in the prognosis of OS, we constructed a classifier containing 12 IR-lncRNAs and 3 IRGs for overall survival by using the least absolute shrinkage and selection operation (LASSO) Cox regression. Based on the risk score calculated by the classifier, the samples were divided into low- and high-risk groups. We further investigated the TME of the OS samples by Estimation of STromal and Immune cells in MAlignant Tumor tissues using Expression (ESTIMATE) data and Cell Type Identification by Estimating Relative Subsets of RNA Transcripts (CIBERSORT) algorithms. Finally, we explore the potential therapeutic small molecular drugs for the OS patients at high risk. Our results demonstrate that the IRGs and IR-lncRNAs–based classifier could be used as a reliable prognostic predictor for OS survival.

## Materials and Methods

### Data Source and Preprocessing

**Figure 1:**
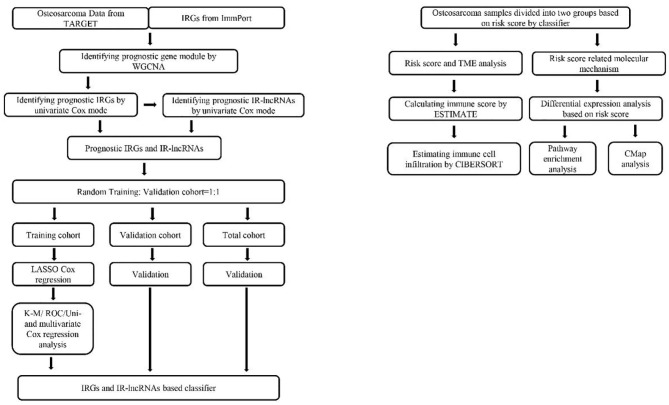
Flowchart of the study involved in construction of IRGs and IR-lncRNAs–based prognostic classifier. IRGs, immune related genes; TARGET, Therapeutically Applicable Research to Generate Effective Treatments; ROC, receiver operating characteristic; K-M, Kaplan–Meier; ESTIMATE, Estimation of STromal and Immune cells in MAlignant Tumor tissues using Expression data; WGCNA, weighted gene coexpression network analysis; TME, tumor microenvironment; cMap, connectivity map; CIBERSORT, Cell Type Identification by Estimating Relative Subsets of RNA Transcripts.

The bioinformatics analysis was conducted following the procedure presented in Figure 1. An RNA-seq data set and the corresponding clinical parameters of OS tissues (*n* = 88) were downloaded from TARGET (Therapeutically Applicable Research to Generate Effective Treatments) (https://ocg.cancer.gov/programs/target). The clinical characteristics of the 88 included samples are summarized in Table 1. The OS patients with complete outcome data and RNA-seq data were included in the subsequent analysis.

**Table 1:** Clinical characteristics of the osteosarcoma patients in TARGET database.

**Parameters**	**Number**	**Ratio (%)**
**Age (y)**		
<16	52	59.1
>16	36	40.9
**Gender**		
Male	51	58.0
Female	37	42.0
**Race**		
White	52	59.1
Asian	7	8.0
Black	7	8.0
Unknown	22	24.9
**Metastasis**		
Positive	22	25
Negative	66	75
**Progression**		
Positive	15	17.0
Negative	24	27.3
Unknown	49	55.7
**Survival status**		
Alive	57	64.8
Death	29	33.0
Unknown	2	2.2

A total of 1,811 IRGs were obtained from Immport Shared Data (https://www.immport.org/shared/home).

### Weighted Gene Co-expression Network Construction and Interesting Module Detection

Weighted gene co-expression network construction and module identification of all IRGs in the OS data set were performed following the protocols of weighted gene co-expression network analysis (WGCNA) (25), described previously (26). Briefly, every pairwise gene–gene relationship was calculated by a gene coexpression similarity in the first step. Then, a “soft” power adjacency function was utilized to construct the adjacency matrix and topological overlap matrix (TOM). “Gene modules,” groups of genes that have high topological overlap, were identified using hierarchical clustering with a dissimilarity measure (1-TOM).

The correlations between modules and clinical features were identified by Pearson correlation tests to identify clinically meaningful modules. The modules that exhibited a high correlation with prognostic features were selected as interesting modules to be further studied.

### Identification of Prognostic IRGs and IR-lncRNAs

We conducted a univariate Cox regression for all IRGs in interesting modules and identified the genes with *P* < 0.05 as prognostic IRGs. Afterward, we conducted Pearson correlation tests between prognostic genes and all lncRNAs of the OS patients; correlation ≥0.6 was identified as IR-lncRNAs. Following this, we conducted a univariate Cox regression for all IR-lncRNAs and identified the lncRNAs with *P* < 0.05 as prognostic IR-lncRNAs.

### Establishment of Prognostic Classifier

We applied the LASSO Cox regression analysis for all prognostic IRGs and IR-lncRNAs to select the most useful prognostic biomarkers and construct the survival-predicting classifier. The prognosis risk scores were calculated based on a formula as follows:

$$\begin{array}{l} \text{Risk score}=\Sigma \text{ genes }(\text{or lncRNAs}){\;}_{\text{Cox coefficient}}\\ \;(\text{or lncRNAs}){\;}_{\text{expression levels}} \end{array}$$

Based on the cutoff of the median risk score, OS patients were divided into low- and high-risk groups. The predictive ability of the model for the training and validation cohort, which randomly split at a 1:1 ratio, as well as the total cohort, was evaluated by the Kaplan–Meier log-rank test. In addition, the application value of the model was tested by using receiver operating characteristic (ROC) curve analysis, and by univariate and multivariate Cox regression analysis.

### Estimation of Immune Score

ESTIMATE was conducted to investigate the TME of OS and explore its correlation with IRGs and IR-lncRNAs–based classifier. ESTIMATE was designed to calculate scores for reflecting the levels of infiltrating immune cells and stromal cells within the TME based on the specific gene expression signatures of immune and stromal cells (27). Based on the cutoff of the median immune score, OS patients were divided into two groups. Besides, Kaplan–Meier method was also applied to assess the relationship between the immune score and the overall survival of OS patients.

### Estimation of Immune Cell Infiltration

In order to further explore the relationship between the classifier and immune cell infiltration, the CIBERSORT algorithm was used to estimate the fraction of 22 immune cell types in the OS samples from gene expression data. Samples with a CIBERSORT output of *P* < 0.05 were considered to be eligible for further analysis. The Wilcoxon rank-sum test was used to identify the immune cells, which had significant differences in the proportion between low- and high-risk groups. Besides, Kaplan–Meier method was also applied to assess the relationship between the infiltrating of immune cells and the overall survival of OS patients.

### Identification of Differentially Expressed Genes and Pathway Enrichment Analysis

The “edgeR” package of R was used to detect the differentially expressed gene (DEGs) between high- and low-risk samples. We set |log_2_ fold change (FC)| ≥1 and *P* < 0.05 as the cutoff criteria. The volcano plot was drawn through the “gplots” package of R. Pathway enrichment analysis of DEGs, including KEGG pathway, Reactome pathway, and PANTHER pathway, was conducted by KOBAS 3.0 database (http://kobas.cbi.pku.edu.cn/anno_iden.php).

### Identification of Potential Small Molecule Drugs

We submitted the DEGs of |log_2_FC| ≥2 into the CMap (Connectivity map) (https://portals.broadinstitute.org/cmap), a database of small-molecule drugs, gene expression profiles, and diseases, which is based on the differential gene expression of human cells treated with small-molecule drugs. An enrichment score representing similarity is finally calculated. The positive connectivity score illustrates that the drug is capable of increasing the risk of death of OS patients. On the contrary, the negative link score indicated that the drug is able to reduce the risk of death. The drugs with negative connectivity score indicated potential therapeutic value. Two-dimensional diagrams of these candidate molecular drugs were obtained in Pubchem database (https://pubchem.ncbi.nlm.nih.gov/).

### Statistical Analysis

All statistical analyses were performed by SPSS version 22.0, IBM Corp., Armonk, NY, United States and R version 3.6.1 software. The correlation between risk score and clinicopathological characteristics was analyzed by the χ^2^ test. The unpaired *t*-test was used to estimate the statistical significance for normally distributed variables of the two groups. Wilcoxon rank-sum test was used to estimate the statistical significance for non–normally distributed variables of the two groups. All statistical tests were two-tailed, with a value of *P* < 0.05 considered statistically significant.

## Results

### Coexpression Network Construction and Interesting Module Detection

**Figure 2:**
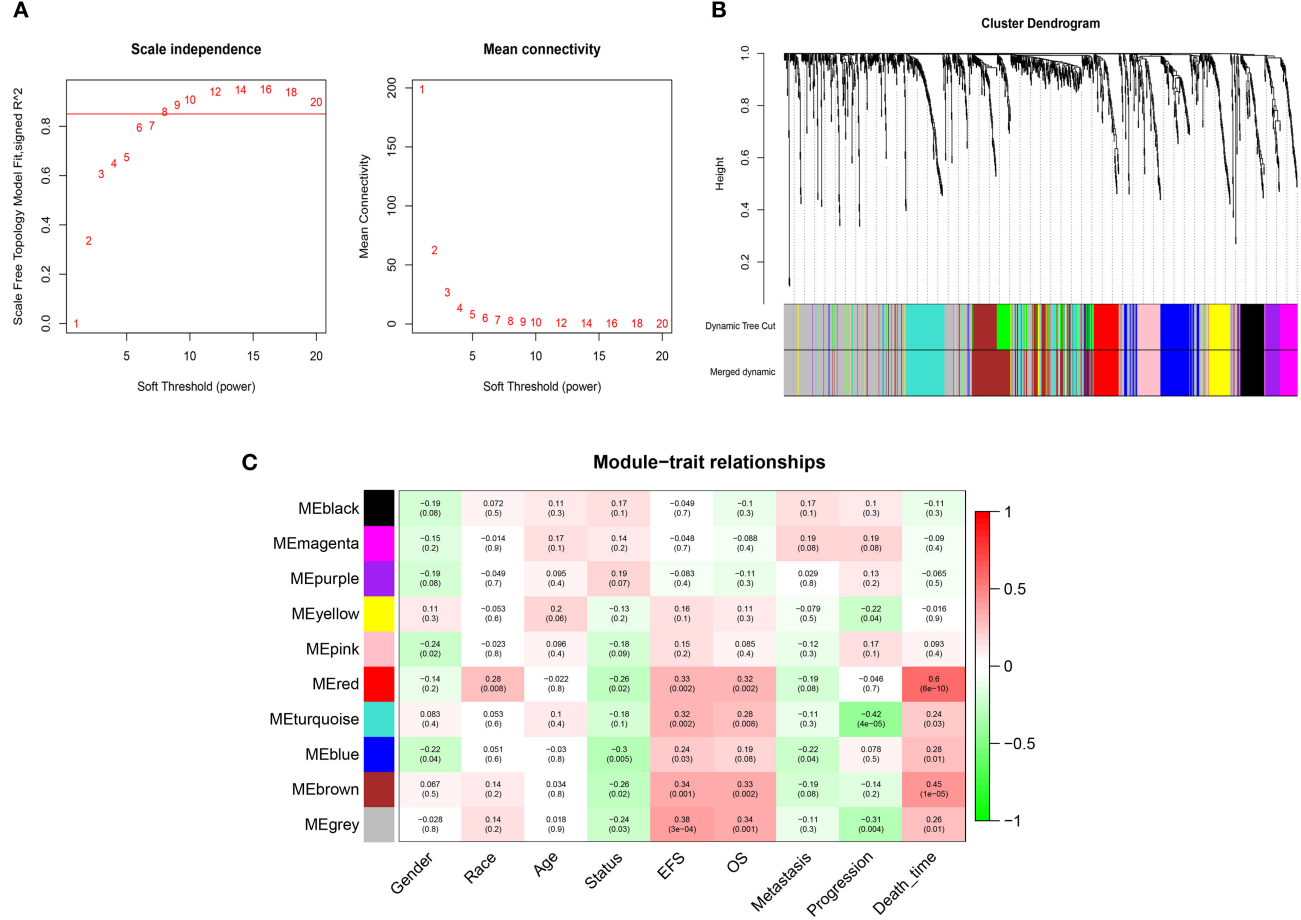
WGCNA network and module detection. **(A)** Selection of the soft-thresholding powers. Power 8 was chosen because the fit index curve flattened out upon reaching a high value (>0.85). **(B)** Cluster dendrogram and module assignment for modules from WGCNA. The colored horizontal bar below the dendrogram represents the modules. **(C)** Correlation matrix for eigengene values and clinical features. Each cell includes the corresponding correlations and the *p*-values.

WGCNA was performed on 1,222 IRGs in the 88 OS samples. After removing one outlier sample, the connectivity between the genes in the gene network formed a scale-free network distribution when the soft-threshold power β was set to 8 (Figure 2A). Then, 10 coexpressed modules were identified and represented by different colors. The “gray” module was reserved for genes identified as not coexpressed (Figure 2B). The correlations between modules and clinical features, such as gender, race, age, EFS (event-free survival), overall survival, metastasis, progression, and death time were calculated. The red module was highly correlated with EFS (*r* = 0.33, *P* = 0.002), overall survival (*r* = 0.32, *P* = 0.002), and death time (*r* = 0.6, *P* = 6 × 10^−6^), and brown module was highly correlated with EFS (*r* = 0.34, *P* = 0.001), overall survival (*r* = 0.33, *P* = 0.002), and death time (*r* = 0.45, *P* = 6 × 10^−5^) (Figure 2C). Thus, the red and brown modules were selected as interesting modules to be studied in subsequent analyses.

### Identification of Prognostic IRGs and IR-lncRNAs

Eighty-six samples with complete survival data were included in the survival analysis. Univariate Cox regression analyses for all IRGs in red (*n* = 62) and brown (*n* = 180) modules were conducted (Supplementary Table 1) and identified 68 genes with *P* < 0.05 as prognostic IRGs (Supplementary Figure 1A). Afterward, 1,591 IR-lncRNAs were identified of correlation ≥0.6 with prognostic IRGs (Supplementary Table 2). Besides, 129 prognostic IR-lncRNAs were identified with *P* < 0.05 of univariate Cox regression analysis (Supplementary Figure 1B).

### Establishment of Prognostic Classifier

**Figure 3:**
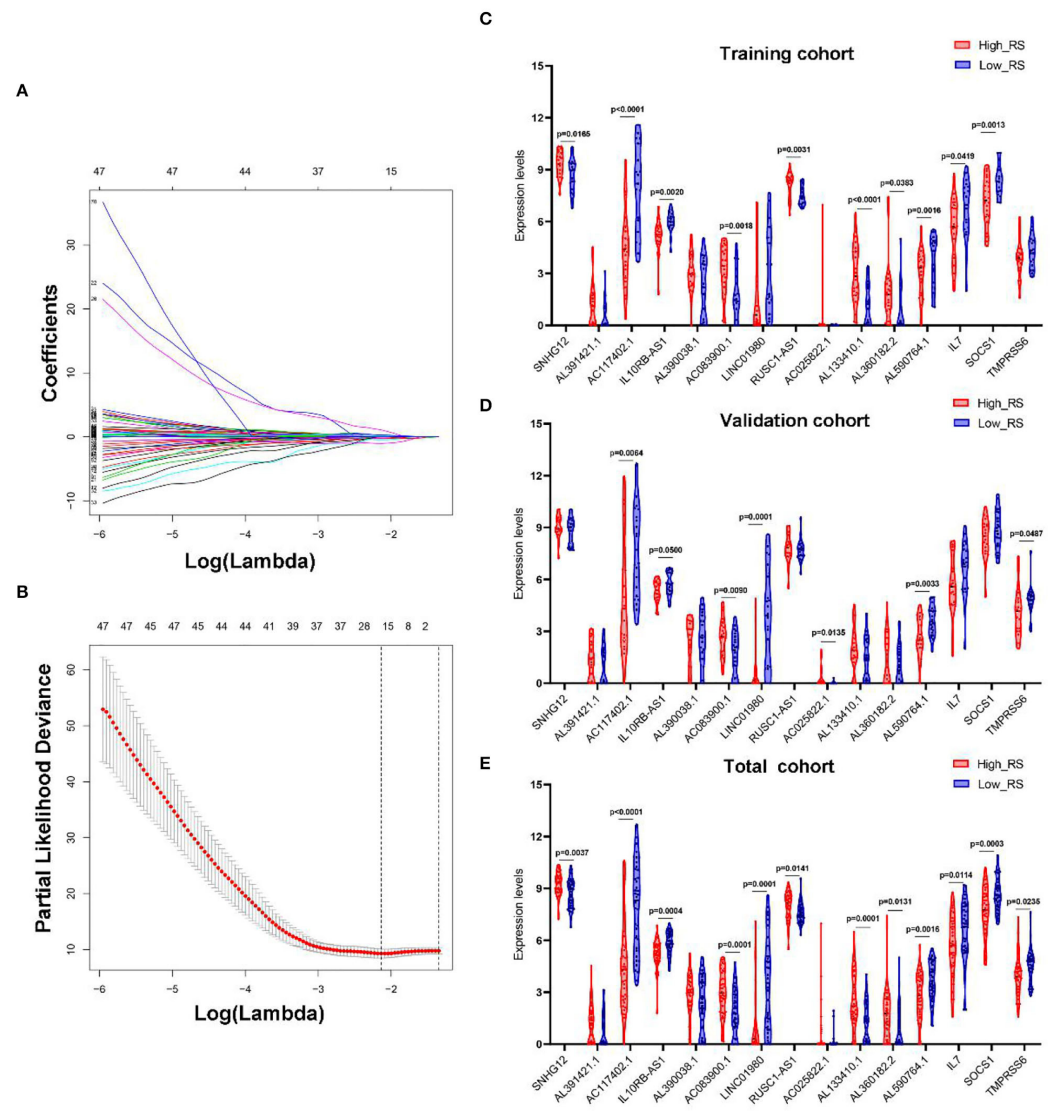
Construction of IRGs and IR-lncRNAs–based prognostic classifier. The results of the LASSO Cox regression suggested that all 15 mRNAs and lncRNAs were essential for the classifier **(A,B)**. The expression levels of all 15 biomarkers of the classifier in high- and low-risk group from the training **(C)**, validation **(D)**, and total **(E)** cohorts. RS, risk score.

LASSO Cox regression analysis was conducted to select the most useful prognostic biomarkers for constructing the prognostic-predicting classifier base on the training cohort (Figures 3A,B). A total of 12 lncRNAs (SNHG12, AL391421.1, AC117402.1, IL10RB-AS1, AL390038.1, AC083900.1, LINC01980, RUSC1-AS1, AC025822.1, AL133410.1, AL360182.2, and AL590764.1) and 3 mRNAs (IL7, SOCS1, and TMPRSS6) were identified as the most useful prognostic biomarkers (Table 2).

**Table 2:** The IRGs and IR-lncRNAs in the prognostic classifier associated with OS in the TARGET data set.

**Type**	**Symbol**	**Univariate cox regression analysis**			**LASSO**
		**HR**	**95% CI**	***P***	**Coefficient**
lncRNAs	SNHG12	2.024	1.213–3.376	0.007	0.953
	AL391421.1	1.553	1.134–2.127	0.006	0.116
	AC117402.1	0.775	0.679–0.885	0.000	−0.279
	IL10RB-AS1	0.421	0.282–0.630	0.000	−1.01
	AL390038.1	1.352	1.037–1.762	0.026	0.031
	AC083900.1	1.845	1.349–2.524	0.000	0.277
	LINC01980	0.739	0.599–0.912	0.005	−0.239
	RUSC1-AS1	2.501	1.462–4.280	0.001	0.267
	AC025822.1	1.405	1.118–1.765	0.004	0.057
	AL133410.1	1.646	1.297–2.088	0.000	0.106
	AL360182.2	1.442	1.180–1.763	0.000	0.150
	AL590764.1	0.604	0.459–0.796	0.000	−0.052
mRNAs	IL7	0.715	0.580–0.881	0.002	−0.032
	SOCS1	0.582	0.437–0.775	0.000	−0.487
	TMPRSS6	0.579	0.400–0.838	0.004	−0.203

The risk scores were calculated using the formula mentioned previously; patients in every cohort were further divided into high- and low-risk groups with the median risk score as the cutoff value. And the expression levels of every biomarker in different groups were analyzed (Figures 3C–E).

### Correlation Between Classifiers and Clinicopathologic Characteristics

As shown in Table 3, all the clinical characteristics (age, gender, race, metastasis, and progression) showed no significant differences between the high- and low-risk groups in the training and validation cohort. However, metastasis showed significant difference between the two groups in the total cohort. Patients with metastasis were inclined to have a higher risk score.

**Table 3:** Correlations between risk score of the immune-related genes and lncRNAs-based classifier with clinicopathological characteristics in training cohort, validation cohort and total cohort.

**Parameters**	**Training cohort (*****n*** **=** **43)**				**Validation cohort (*****n*** **=** **43)**				**Total cohort (*****n*** **=** **86)**			
	**High risk**	**Low risk**	**χ^**2**^**	***P***	**High risk**	**Low risk**	**χ^**2**^**	***P***	**High risk**	**Low risk**	**χ^**2**^**	***P***
**Age (y)**			0.352	0.553			0.011	0.916			0.047	0.829
<14	13	12			7	7			19	20		
>14	11	7			14	15			24	23		
**Gender**			0.266	0.206			0.011	0.916			0.047	0.828
Male	12	8			14	14			24	25		
Female	12	11			7	8			19	18		
**Race**			0.687	1.000			0.386	1.000				
White	15	16			8	12			22	29	0.618	0.904
Asian	1	1			2	3			3	4		
Black	2	1			2	2			4	3		
**Metastasis**			1.063	0.302			3.376	0.066			5.103	**0.024**
Positive	7	3			8	3			15	6		
Negative	17	16			13	19			28	37		
**Progression**			1.21	0.271			0.099	0.753			1.269	0.26
Positive	7	1			3	4			10	5		
Negative	3	2			9	9			11	12		

*The bold value means statistically significant*.

### Prognostic Value of Classifiers for Assessing Overall Survival

**Figure 4:**
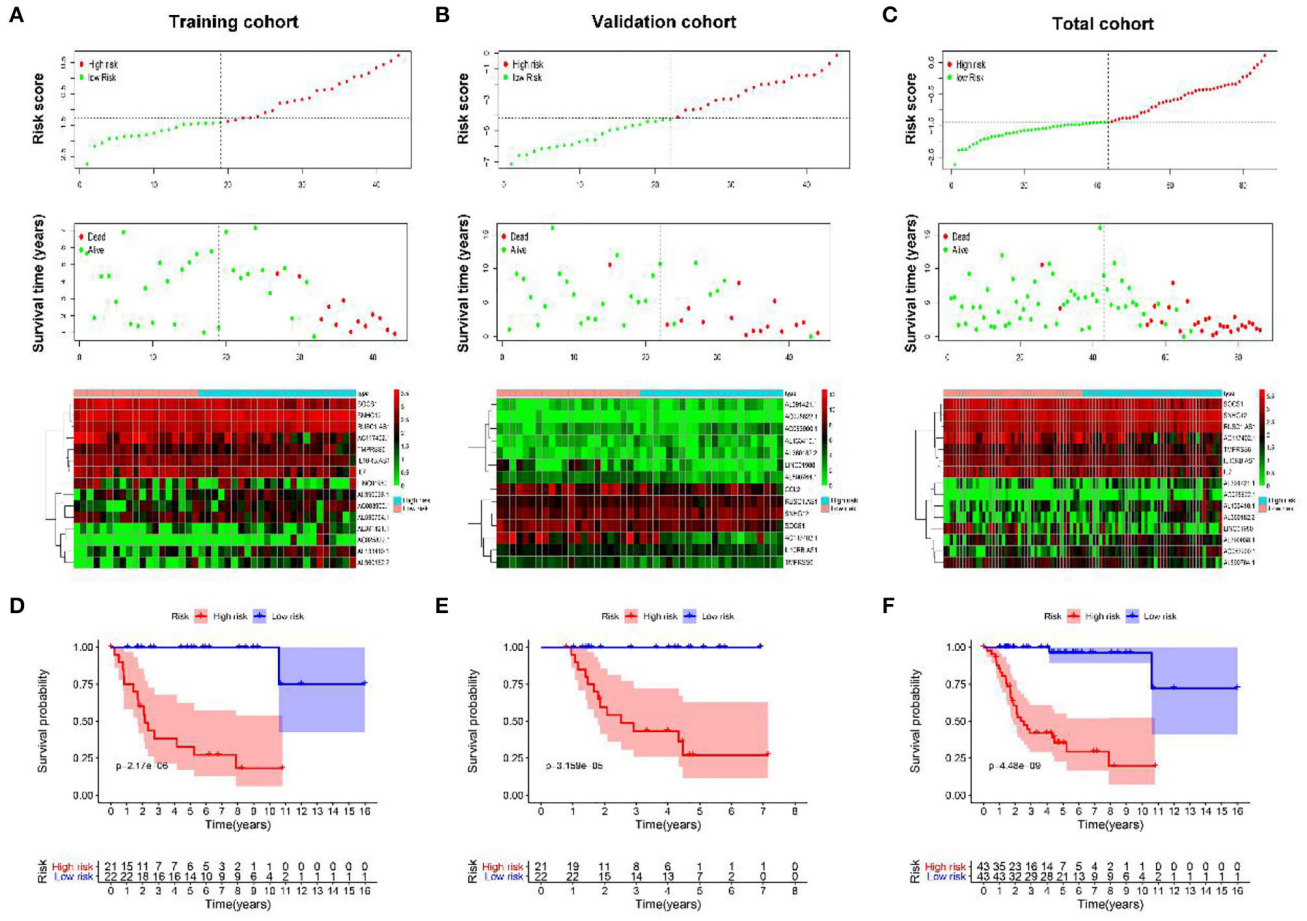
The prognostic value of IRGs and IR-lncRNAs–based classifier. The distribution of patients' risk score, survival state, and expression of all 15 biomarkers of the classifier in high- and low-risk group from the training **(A)**, validation **(B)**, and total **(C)** cohorts. Kaplan–Meier survival analysis of overall survival between high- and low-risk patients from the training **(D)**, validation **(E)**, and total **(F)** cohorts.

As shown in Figures 4A–C, with the increase of risk score, the survival time of patients is decreased, and almost all the dead patients were enrolled in the high-risk group.

To further assess the prognostic value of the classifier, Kaplan–Meier test was conducted. As shown in Figures 4D–F, patients in high-risk group had significantly unfavorable prognosis.

**Figure 5:**
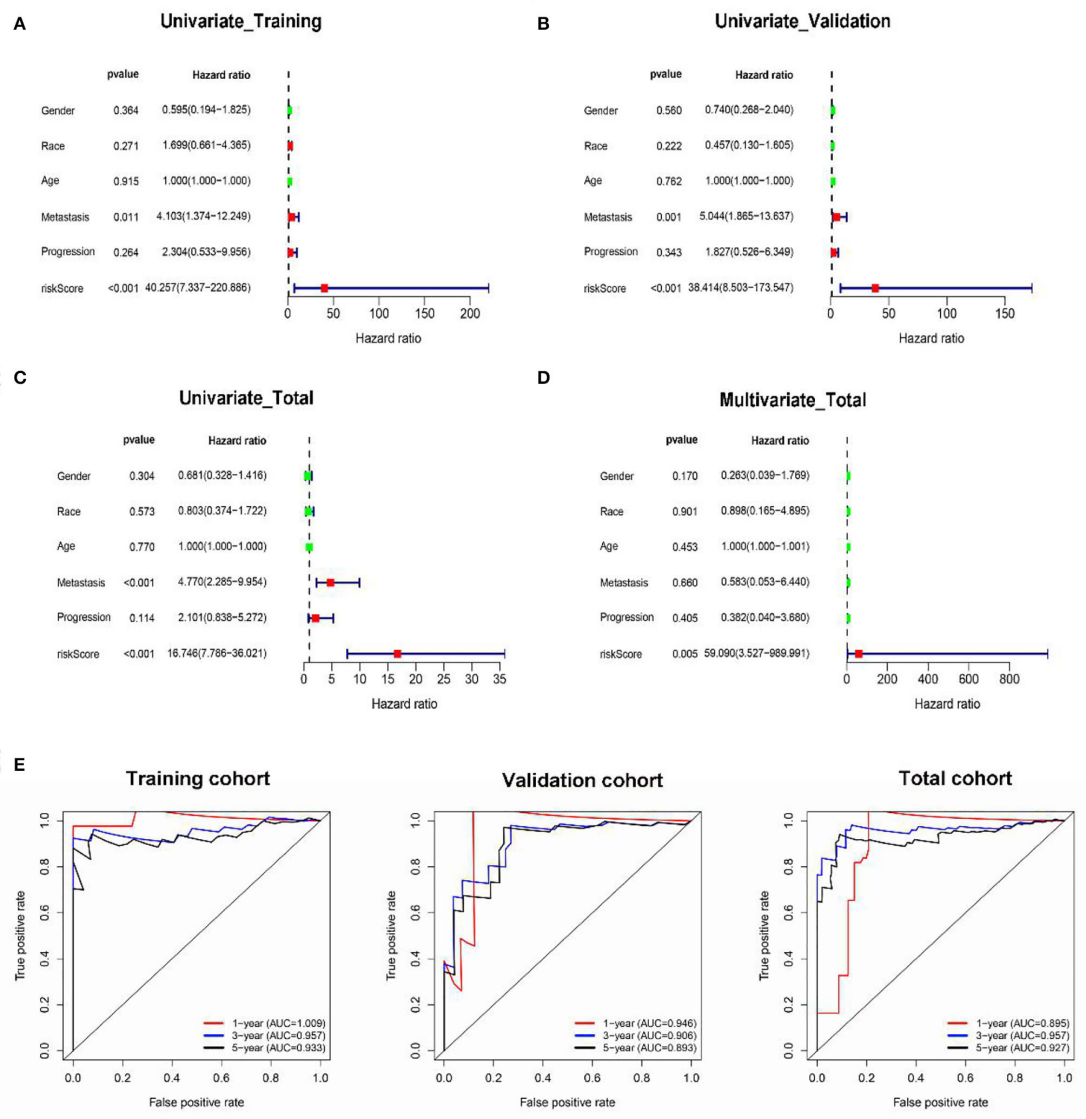
The prognostic value of the classifier. Univariate and multivariate Cox regression analyses of the classifier with overall survival in the training **(A)**, validation **(B)**, and total **(C,D)** cohorts. The time-dependent ROC for 1-, 3-, and 5-years overall survival predictions for the classifier in training, validation, and total cohorts **(E)**.

Besides, the results of univariate Cox regression analysis in training, validation, and total cohorts further validated the prognostic value of classifier (Figures 5A–C). Moreover, multivariate analysis in the total cohort suggested that the classifier was an independent risk factor of survival for OS patients (Figure 5D).

In addition, in the time-dependent ROC curve analysis, the areas under the curve for overall survival in the first, third, and fifth year were 1.009, 0.957, and 0.933, respectively, in the training cohort (Figure 5E), 0.945, 0.963, and 0.927, respectively, in the validation cohort; and 0.875, 0.956, and 0.927, respectively, in the total cohort. Moreover, the prediction capability of the classifier was superior to metastasis, which may be a major risk factor for tumor prognosis as reported by previous studies (Supplementary Figure 2).

The results above indicate that the IRGs and IR-lncRNAs–based classifier provided a useful prognostic tool with clinical value for appropriately categorizing patients with OS.

### Patients With Low Risk Scores Correlated With High Immune Scores

**Figure 6:**
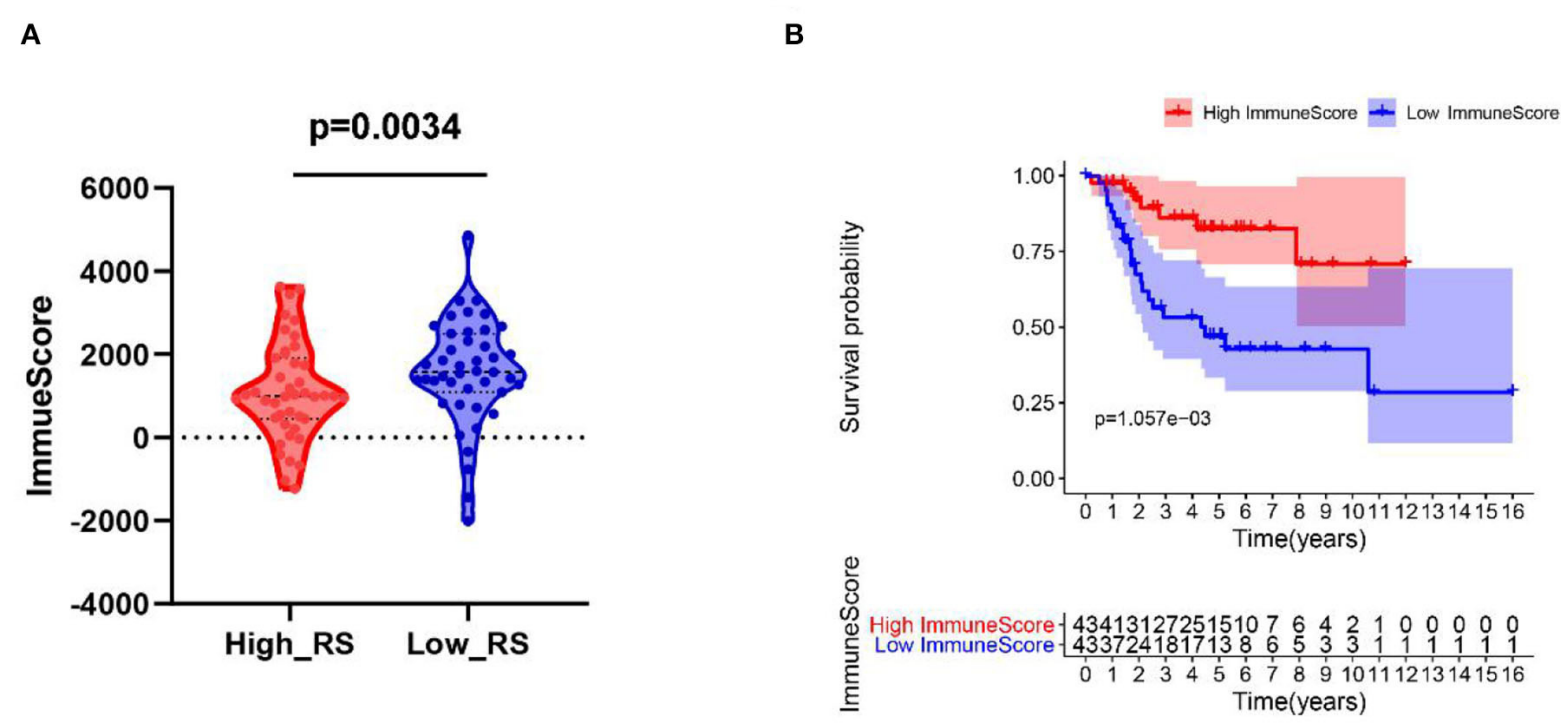
**(A)** High-risk score correlated with low immune score. **(B)** Kaplan–Meier survival analysis of overall survival between high and low immune score. RS, risk score.

As shown in Figure 6A, patients with low risk scores were related to high immune scores. Moreover, patients with high immune scores were correlated with better prognosis (Figure 6B).

### The Landscape of Immune Infiltration in OS

**Figure 7:**
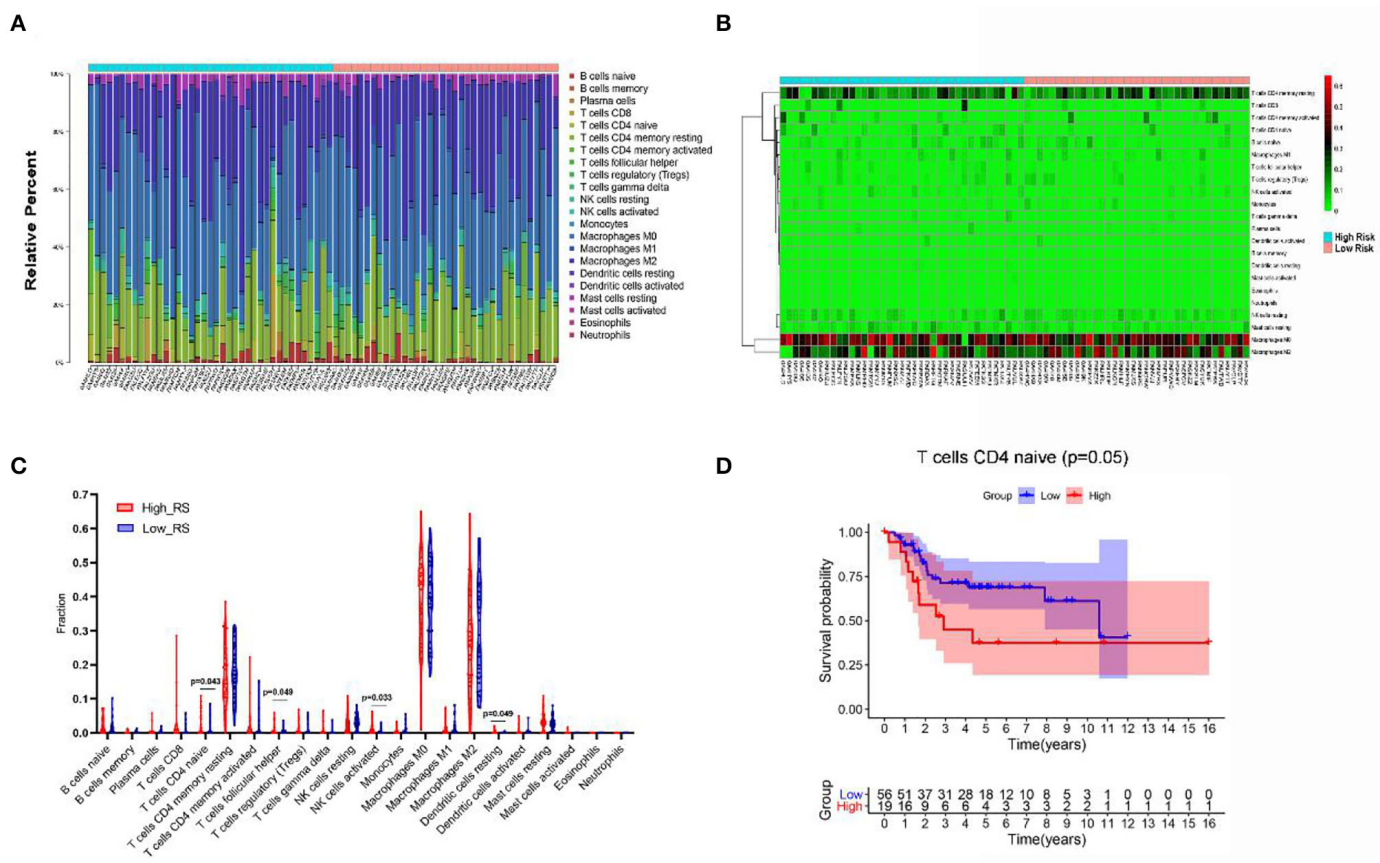
The composition **(A)** and heat map **(B)** of immune cells estimated by CIBERSORT algorithm in OSs. **(C)** The comparison of the fractions of immune cells between high- and low-risk group. **(D)** Kaplan–Meier survival analysis of overall survival between high and low level of infiltrating T-cell CD4 naive.

As shown in Figure 7A, we created a bar plot to demonstrate the proportion of 22 immune cells in each sample, which revealed that the five immune cells with the highest proportion in OS were M0 macrophages (38.6%), M2 macrophages (27.8%), T-cell CD4 memory resting (17.2%), mast cells resting (3.0%), and natural killer (NK) cells resting (2.9%). Then, we plotted the heat map of 22 immune cells in Figure 7B.

Additionally, Wilcoxon rank-sum test was used and revealed that the fractions of T-cell CD4 naive (*P* = 0.043), T-cell follicular helper (*P* = 0.049), dendritic cells resting (*P* = 0.049), and NK cells activated (P = 0.033) varied significantly between high- and low-risk-score patients (Figure 7C).

Besides, Kaplan–Meier analysis revealed that patients with low proportion of T-cell CD4 naive are associated with better overall survival (*P* = 0.05) (Figure 7D).

### Screening for DEGs

**Figure 8:**
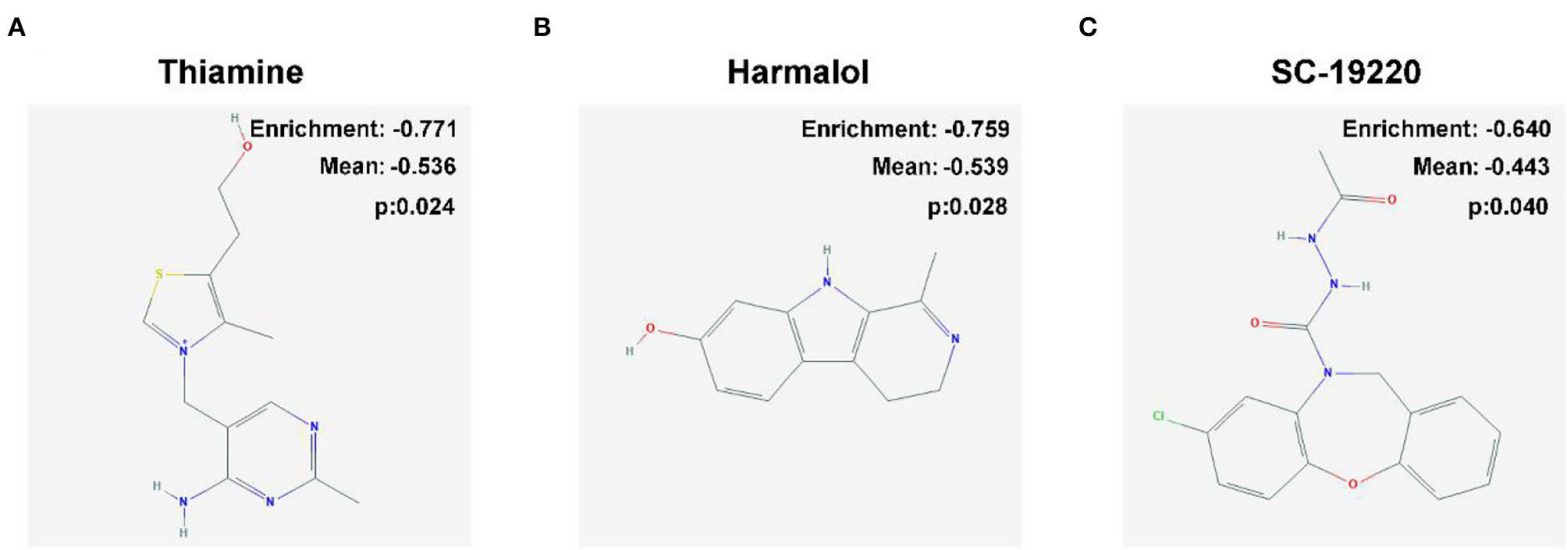
Two-dimensional diagram of the three most significant drugs. **(A)** Thiamine, **(B)** harmalol, and **(C)** SC-19220.

A total of 1,135 DEGs, including 316 upregulated genes and 819 downregulated genes, were identified in the high-risk group, compared with the low-risk group (Figure 8A). We further performed pathway enrichment analysis for these DEGs. As shown in Figure 8B, the upregulated genes mainly enriched in the pathways of class A/1 (rhodopsin-like receptors), peptide ligand-binding receptors, GPCR ligand binding, GPCR downstream signaling, activation of C3 and C5, neuroactive ligand–receptor interaction, diseases of metabolism, signal transduction, inflammation mediated by chemokine and cytokine signaling pathway, and cell–cell communication. However, the downregulated genes mainly enriched in the pathways of transmembrane transport of small molecules, GPCR ligand binding, GPCR downstream signaling, class A/1 (rhodopsin-like receptors), neuroactive ligand–receptor interaction, starch and sucrose metabolism, retinol metabolism, drug metabolism–cytochrome P450, biological oxidations, and amino acid conjugation (Supplementary Figure 3).

### Potential Small Molecule Drugs

We uploaded 404 DEGs of |log_2_FC| ≥2, consisting of 300 downregulated genes and 104 upregulated genes, into the CMap network tool. Among these highly significant correlated molecules, thiamine, harmalol, and SC-19220 were most negatively correlated with high-risk OS patients (Figure 8). They all might have the potential therapeutic effects on OS patients with high risk.

## Discussion

Osteosarcoma is the most common malignancy of bone and is characterized by highly aggressive and metastasis, which results in the very poor prognosis of patients (2). Thus, the identification of effective biomarkers for OS-specific prognoses is urgently needed to improve the management for patients. Taking into account the importance of the immune system in the progression of cancers and the highly specialized immune environment of bone (3, 28), it is essential to find out immune-related biomarker for the prognosis of OS patients, which may also play a significant role in immunotherapy.

In the present study, we constructed a prognostic classifier for OS by combining IRGs and IR-lncRNAs for the first time. A 12 IR-lncRNAs– and 3 IRGs-based classifiers for overall survival were constructed and validated to optimize the predictive ability of prognosis for OS patients. The results indicated that the classifier could successfully divide OS patients into high- and low-risk groups with significant differences in overall survival in the training cohorts. The prognostic value of the classifier was also confirmed in the validation cohort and the total cohort, indicating the repeatability and practicability of the IRGs- and IR-lncRNAs–based classifiers for the prognostic prediction for overall survival. Besides, the prediction capability of the classifier was superior to metastasis, which may be a major risk factor for tumor prognosis as reported by previous studies (29).

Among these 15 IR biomarkers, SNHG12, AL391421.1, AL390038.1, AC083900.1, RUSC1-AS1, AC025822.1, AL133410.1, and AL360182.2 were risk-associated, whereas AC117402.1, IL10RB-AS1, LINC01980, AL590764.1, IL7, SOCS1, and TMPRSS6 were protective (Table 1). Although some of the IR-lncRNAs in our classifier have not been functionally annotated and completely clarified, other biomarkers used in our classifiers have been explored. Previous studies showed that SNHG12 promoted tumorigenesis and metastasis in OS through upregulating NOCTH2 by sponging miR-195-5p (30). IL7 treatment promotes immune reconstitution significantly and improves the overall survival of pediatric sarcoma patients (31). SOCS1 acts as a cancer suppressor by promoting apoptosis and suppressing the metastasis of OS (32). Low expression of TMPRSS6 is related to the triple-negative and high grade of breast cancer (33). The upregulation of LINC01980 promotes tumor growth of esophageal squamous cell carcinoma (34). RUSC1-AS1 promotes the proliferation of breast cancer by inhibiting KLF2 and CDKN1A, which may serve as a potential hallmark for breast cancer (35). Given their strong relevance to prognosis, these genes should be explored in the future, especially in relation to OS.

Recently, many studies have demonstrated that tumor-infiltrating immune cells were associated with prognosis (36, 37). ESTIMATE algorithm is a simple method to predict the infiltration of immune cells by analyzing specific gene expression signature of immune cells and outputting immune scores (27). Previous ESTIMATE analyses have shown that immune cell infiltration is associated with prognosis in patients with various types of tumors (38, 39). In the present study, we found that the risk score based on the classifier negatively correlated with the immune score. Besides, patients with high immune scores have a favorable prognosis, indicating that immune cell infiltration of the TME could have a beneficial impact on prognosis. To further investigate the infiltration of immune cells, we conducted CIBERSORT analysis to illustrate the types of infiltrating cells. T-cell CD4 naive, T-cell follicular helper, dendritic cells resting, and NK cells activated were found significantly lower in the low-risk group. Besides, a low proportion of T-cell CD4 naive related to a better prognosis. Previous study revealed that tumor-infiltrating naive CD4 T cells are the important source of tumor-infiltrating regulatory T cells, which suppress the antitumor function of effector T cells and NK cells (40). Inhibiting the recruitment of T-cell CD4 naive into tumors reverses immunosuppression in breast cancer (41). Moreover, previous studies have shown that tumor-infiltrating T-cell follicular helper produced IL4 to suppress antitumor immunity by inducing myeloid cells to differentiate into M2 macrophages (42). Thus, infiltrating of T-cell CD4 naive and T-cell follicular helper may play important roles in the progression of OS, which will be well worth investigating.

Despite numerous attempts were done to improve the prognosis of OS, the outcome has remained unchanged over the past 3 decades (43). Herein, we identified three small molecules, thiamine, harmalol, and SC-19220, with potential therapeutic efficacy against OS. Thiamine (vitamin B_1_) is a coenzyme for transketolase, pyruvate dehydrogenase, and α-ketoglutarate dehydrogenase complexes, which plays fundamental roles in various intracellular metabolism processes (44), as well as the regulation of immune system (45). The role of thiamine in immune responses has been demonstrated in the brain that thiamine plays significant anti-inflammation roles in inhibiting the expression of proinflammatory factors (cyclooxygenase-2, IL1, IL6, and TNF) and suppressing the CD40L-mediated immune and inflammatory responses (46). Current views on the role of thiamine in tumorigenesis are controversial (47). Some studies showed that thiamine was much higher in tumor tissues than in adjacent normal tissues (48), and a low dose of thiamine supplementations promoted cancer growth (49), suggesting that antithiamine is a potential way for cancer therapy. On the other hand, some studies showed that a high dose of thiamine reduced cell viability in breast cancer cells, but not in normal breast epithelial cells (50). Thus, the role of thiamine in OS is well worth investigating. Harmalol, a β-carboline alkaloid, presents in several plants such as *Peganum harmala* (51). Previous studies showed that harmalol treatment induced apoptosis of lung and liver cancer cells by activating caspase-8, caspase-3, and p53 (52, 53), indicating a potential antitumorous role of harmalol for OS. However, the role of harmalol on the immune system remains unclear. Prostaglandin E_2_ (PGE_2_) is a bioactive lipid that displays a wide array of biological effects associated with inflammation and cancer (54). Accumulation of PGE_2_ in a cancer cell environment is a marker for the progression of many cancers (55). Blocking PGE_2_ abrogates bladder cancer chemoresistance (56). SC-19220 is a prostaglandin E_2_ antagonist, which showed potent anti-inflammation by suppression cytosolic phospholipase A_2_ (57) and antitumor capacities by promoting tumor cell apoptosis through E-prostanoid 1 suppression (58). Collectively, thiamine, harmalol, and SC-19220 possess high clinical potential worthy of further investigation for the treatment of OS, especially through the mechanisms of modulating the immune system.

Inevitably, the present study has some innate limitations that need to be addressed. First, it was a retrospective study based on the publicly online database. Second, the cohort of the current study consisted of only 88 samples, and there is no cohort for validation from other databases. Thus, large-scale, multicenter studies are needed to confirm our results before the IRGs- and IR-lncRNAs–based classifier can be applied in the clinic.

## Conclusion

In our study, we first identified and validated a classifier containing 12 IR-lncRNAs and 3 IRGs with independent prognostic significance for patients with OS. Moreover, our classifier can also provide novel clinical applications for immunotherapies and immune targets for OS. Besides, based on the classifiers, we identified three small molecular drugs with potential therapeutic value for OS treatment.

## Data Availability Statement

Publicly available datasets were analyzed in this study, available from the TARGET database (https://ocg.cancer.gov/programs/target).

## Author Contributions

TZ and YN: design, analysis and interpretation of data, drafting of the manuscript, and critical revision of the manuscript. HX and KC: statistical analysis. YZ: acquisition of data. XC, HL, and JW: critical revision of the manuscript for important intellectual content, administrative support, obtaining funding, and supervision. All authors: read and approved the final manuscript.

## Conflict of Interest

The authors declare that the research was conducted in the absence of any commercial or financial relationships that could be construed as a potential conflict of interest. The reviewer FL declared a shared affiliation, with no collaboration, with the authors to the handling editor at the time of the review.

## Supplementary Material

Supplementary Figure 1 Univariate Cox analysis for the IRGs **(A)** and IR-lncRNAs **(B)**.

Supplementary Figure 2 The time-dependent ROC for 1-, 3-, and 5-years overall survival predictions for the classifier in comparison with clinical features in the training **(A)**, validation **(B)**, and total **(C)** cohorts.

Supplementary Figure 3 DEGs screen and pathway enrichment analysis. **(A)** Volcano plot of DEGs between high and low-risk groups in the total cohort. Pathway enrichment analysis of up- **(B)** and down- **(C)** regulated genes.

Supplementary Table 1 Gene list of red and brown modules.

Supplementary Table 2 List of Immune-related lncRNAs.
