# Supplementary figures and images for: Identification of Immune-Related Prognostic Genes and LncRNAs Biomarkers Associated With Osteosarcoma Microenvironment
Source: Front Oncol. 2020 Jul 24;10:1109. doi: 10.3389/fonc.2020.01109 (PMC7393189; doi:10.3389/fonc.2020.01109)

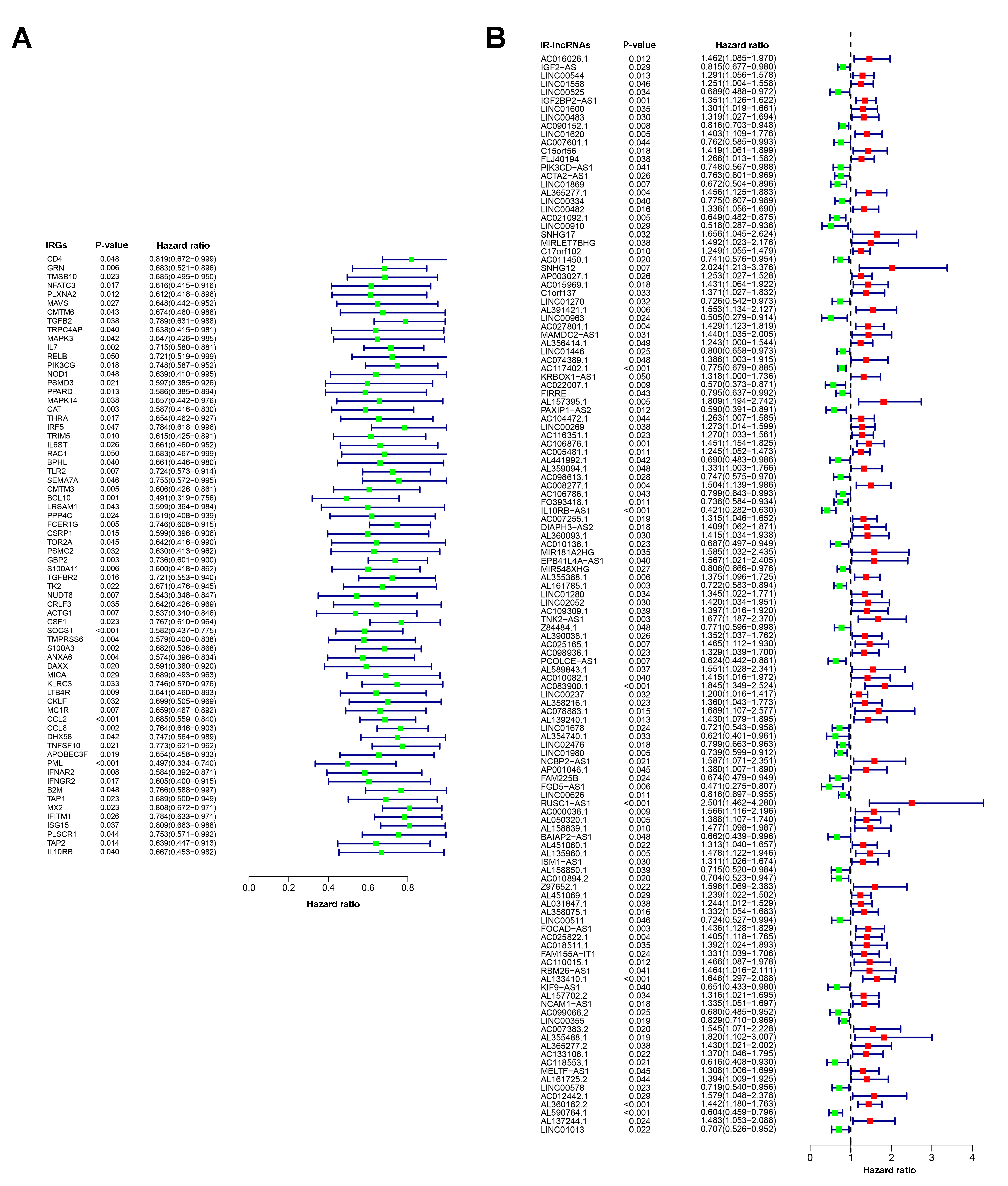

Supplement: Supplementary Figure 1 — Univariate Cox analysis for the IRGs (A) and IR-lncRNAs (B). [file Image_1.TIF]

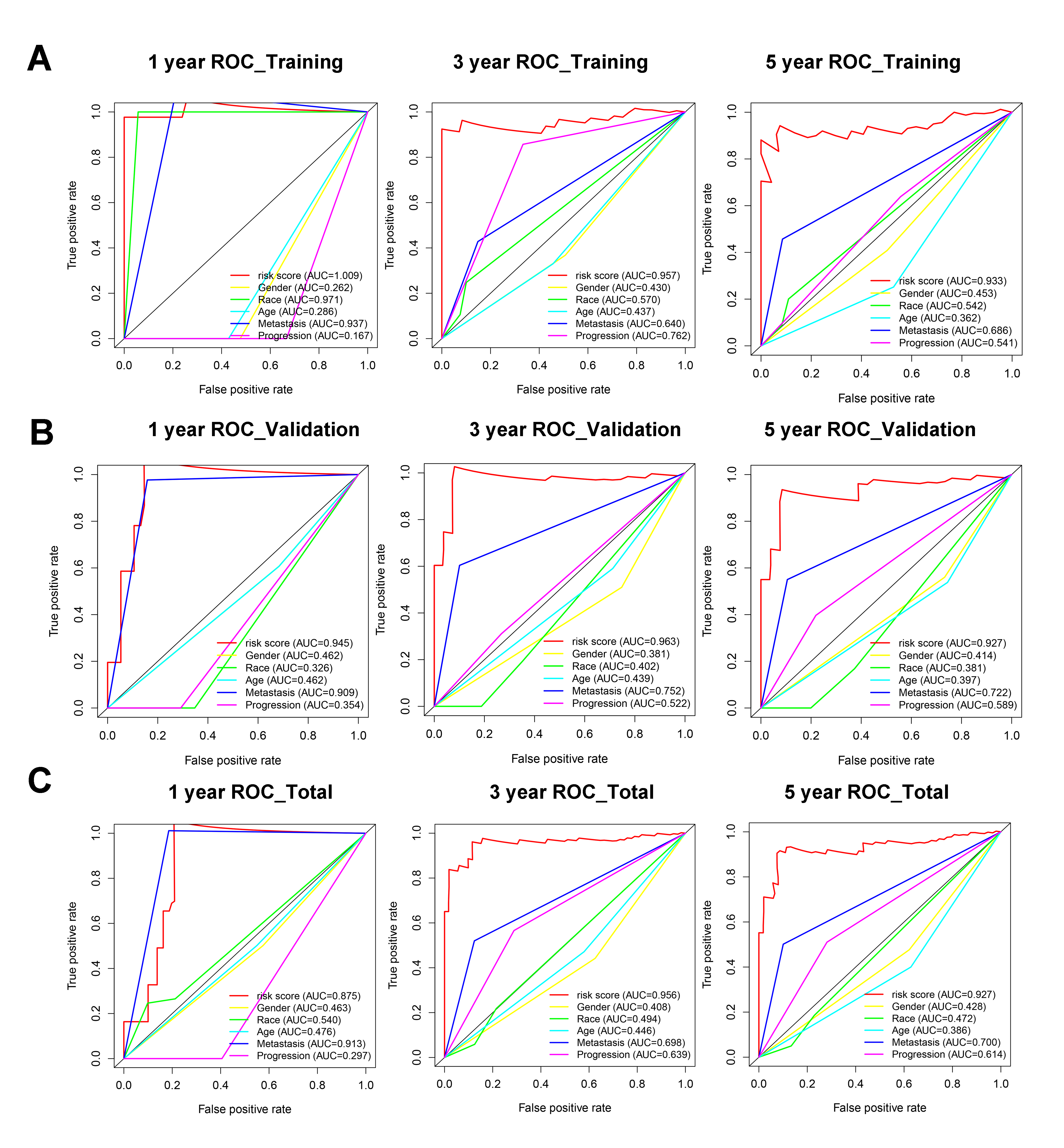

Supplement: Supplementary Figure 2 — The time-dependent ROC for 1-, 3-, and 5-years overall survival predictions for the classifier in comparison with clinical features in the training (A), validation (B), and total (C) cohorts. [file Image_2.TIF]

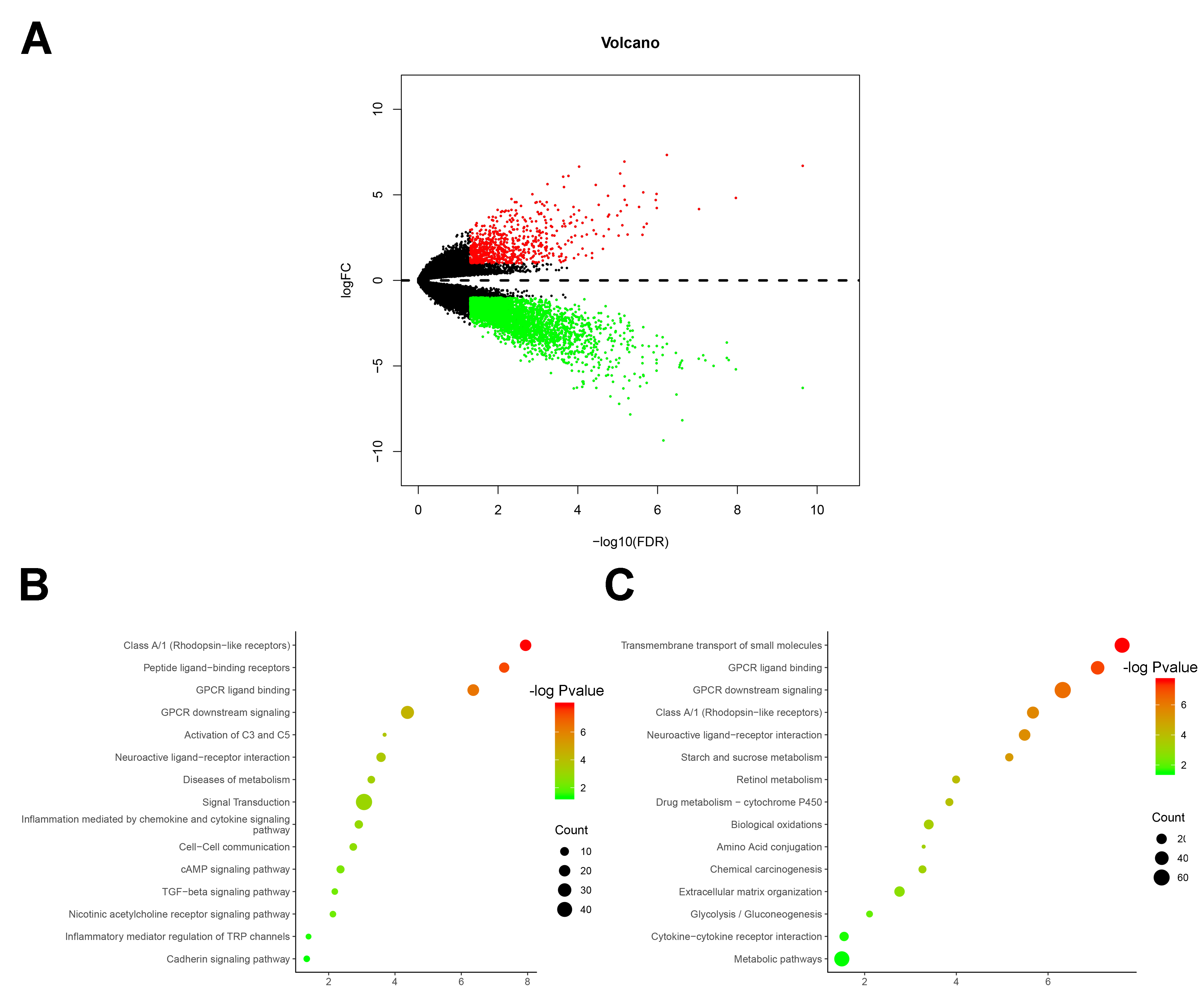

Supplement: Supplementary Figure 3 — DEGs screen and pathway enrichment analysis. (A) Volcano plot of DEGs between high and low-risk groups in the total cohort. Pathway enrichment analysis of up- (B) and down- (C) regulated genes. [file Image_3.TIF]
