# Supplementary material for: Identification of Immune-Related Prognostic Genes and LncRNAs Biomarkers Associated With Osteosarcoma Microenvironment
Source: Front Oncol. 2020 Jul 24;10:1109. doi: 10.3389/fonc.2020.01109 (PMC7393189; doi:10.3389/fonc.2020.01109)
Supplement: Supplementary Table 1 — Gene list of red and brown modules. [file Table_1.DOCX]

| **Immune-related lncRNAs** |
| --- |
| 1ID, LINC01587, AC016026.1, IGF2-AS, DLEU2L, AL591845.1, PINK1-AS, SPG20-AS1, LINC00544, KIAA0087, AC105760.1, DKFZP434K028, LINC00029, FAM182A, C22orf24, H19, AC091132.1, LINC00470, LINC01260, C7orf69, AL355922.1, AC062028.1, LINC01558, LINC00525, LINC00266-1, AL136982.1, PART1, LINC00467, FAM27C, ZFHX2-AS1, TUBA3FP, C1orf147, LINC01931, C2orf48, LINC01116, SNHG16, IGF2BP2-AS1, LINC01600, SMAD5-AS1, C10orf25, ZNF667-AS1, AL357033.1, LINC00483, AC087380.1, AC090152.1, LINC00917, CMAHP, LINC01620, AL512625.1, AC093323.1, TPT1-AS1, MIR31HG, SERHL, PRSS30P, MIR4435-2HG, CCDC13-AS1, AC008080.1, AC013489.1, AC020659.1, SNHG11, C20orf166-AS1, MIR1-1HG, GRIK1-AS1, LRRC75A-AS1, TMEM51-AS1, AC007601.1, LINC00476, C11orf44, C15orf54, LINC01106, AP002986.1, FO393415.1, LINC00302, C10orf111, AL138899.1, AC104129.1, AL033381.1, AC008969.1, C20orf197, TTTY14, C15orf56, LINC00303, MIR7-3HG, TYMSOS, AP000679.1, MRVI1-AS1, LINC00982, LINC01561, C8orf31, DLGAP1-AS1, LINC00469, FLJ13224, FLJ40194, AC021054.1, ZFAS1, CASC2, AC011944.1, AC105206.1, AC025171.1, FAM87B, AL162595.1, AC098864.1, FAM27E5, AL357314.1, LINC01658, AC068473.1, LINC00843, C5orf64, ADORA2A-AS1, LINC00324, AC020907.1, C9orf106, LINC00670, MTUS2-AS1, LINC00311, LINC00174, AC073072.1, AL049647.1, EIF3J-AS1, AP003471.1, FLJ37453, PCBP1-AS1, PIK3CD-AS1, LINC00652, C10orf91, ACTA2-AS1, LINC01869, LINC00304, AC022148.1, C9orf139, LINC02363, WDFY3-AS2, CSNK1G2-AS1, LINC01559, LINC01555, AC105219.1, AC004832.1, FER1L6-AS1, HECW1-IT1, LINC00471, CELF2-AS1, AP003774.1, LINC00301, OGFRP1, AL365277.1, TP53TG1, C17orf77, FAM87A, AC138028.1, LINC00334, LINC01006, PRKCZ-AS1, TSPEAR-AS2, AC138356.1, WT1-AS, LINC01547, AC005037.1, FLJ40288, COL18A1-AS1, AC131971.1, LINC00518, AL021453.1, C11orf72, LINC00315, UMODL1-AS1, AP001062.1, FAM167A-AS1, B3GALT5-AS1, LINC00308, AC000068.1, LINC00482, LINC00313, WASIR1, LINC00158, HDHD5-AS1, LINC01405, LINC00839, AC021092.1, MATN1-AS1, AC016757.1, MIR22HG, KTN1-AS1, C2orf27B, LINC01551, LINC00207, C17orf82, AC092118.1, AC100800.1, TCL6, AC097382.1, LINC00265, PP7080, AC007731.1, AC092171.1, C22orf34, AC010969.1, LINC00319, CYP51A1-AS1, LINC00910, LINC00994, PAX8-AS1, AC069277.1, LINC00943, LINC01164, ANKRD62P1-PARP4P3, AC073349.1, SPATA41, AC079610.1, COLCA1, LINC00523, AC005154.1, C9orf163, AL138767.1, LUADT1, LINC00173, PDXDC2P-NPIPB14P, LINC01560, SNHG17, AC079612.1, CTBP1-AS2, SCOC-AS1, AL360004.1, NPSR1-AS1, AC068631.1, CH17-340M24.3, MIRLET7BHG, LINC00336, RAMP2-AS1, AC090673.1, GATA3-AS1, C17orf102, AC008543.1, C16orf47, AC003975.1, LINC00477, AC068051.1, ATP11AUN, AL157838.1, AC011450.1, AC122129.1, CYYR1-AS1, SNHG12, AC025279.1, AFDN-AS1, AL354714.1, FLVCR1-AS1, AC007920.1, C20orf203, LINC01565, SSBP3-AS1, MUC2, AC013549.1, AL590705.1, AL022323.1, TDRKH-AS1, AL626787.1, C9orf41-AS1, AL445248.1, AC012358.1, AP003027.1, LINC01562, AL355802.1, AL390760.1, LINC01317, AC105020.1, AC015969.1, AL591503.1, LINC00449, AC004988.1, AC009163.1, PDCD4-AS1, AL139390.1, RBMS3-AS2, AP000753.1, Z99756.1, LINC02408, IGBP1-AS1, AC005342.1, LINC00970, AL354919.1, AC144450.1, AC083799.1, LINC00501, LINC01285, IBA57-AS1, AL356123.1, SERTAD4-AS1, C1orf132, C6orf99, LINC00862, AL645568.1, LINC00222, ADAMTSL4-AS1, BVES-AS1, LIN28B-AS1, C1orf137, ATP1A1-AS1, SNHG5, ADD3-AS1, SPATA42, LINC00632, AL354984.1, AL772363.1, ARRDC1-AS1, LINC01270, COL5A1-AS1, SLC12A5-AS1, AL391421.1, LINC00963, AL158151.1, TDRG1, LINC00951, AL162293.1, LINC00619, AP000911.1, PSMB8-AS1, LINC01993, TNRC6C-AS1, LINC01973, AL590644.1, C10orf126, PKP4-AS1, AL133343.1, C9orf170, LINC01854, C1orf195, PSORS1C3, ACOXL-AS1, AC027801.1, LINC01123, LINC01257, AC115618.1, HCG9, C5orf60, AC010624.1, AL133464.1, STARD7-AS1, MAMDC2-AS1, LINC01556, AC008429.1, LINC01291, AL590399.1, ST8SIA6-AS1, AC011484.1, FAM201A, AC021218.1, LINC01545, AC007389.1, ATP6V0E2-AS1, FAM83A-AS1, AP000812.1, LINC02138, AC092384.1, AC134312.1, AC118344.1, LINC01121, LINC02397, DKFZp779M0652, C7orf66, LINC00654, TTLL10-AS1, LINC01602, AL356414.1, AC005863.1, LINC01460, LINC00661, AC007608.1, AL592528.1, AL034376.1, CALML3-AS1, AC013472.1, AC121338.1, CHKB-AS1, AL049775.1, MUC19, LINC01597, AP001043.1, LINC01446, LINC01310, LINC00898, FAM239C, FAM239B, BX890604.1, AC020741.1, ADARB2-AS1, AL359878.1, AC083864.1, LINC01531, DPP9-AS1, LOH12CR2, AC024132.1, LINC00487, C1RL-AS1, AC108134.1, SRRM2-AS1, C21orf62-AS1, AC105345.1, AC074389.1, Z99774.1, AL121823.1, AC006305.1, LINC02346, DUXAP8, AL359555.1, HCP5, HCG27, H1FX-AS1, AC117402.1, KRBOX1-AS1, AC022007.1, THUMPD3-AS1, MIR219A2, AP000346.1, AC003035.1, LINC01089, AL137145.1, EWSAT1, AC093281.1, Z97192.1, AC016747.1, C1orf220, AL021068.1, AL590867.1, Z97192.2, LINC00671, FIRRE, SLX1A-SULT1A3, U73169.1, ZNF337-AS1, LINC01521, LIPE-AS1, AC019080.1, AC007277.1, AL157395.1, LINC02418, LINC02347, PAXIP1-AS2, LINC00887, LINC02026, GCC2-AS1, ST7-OT4, AC010336.1, APTR, VAC14-AS1, LINC00488, KANSL1-AS1, LINC02085, AC068831.1, AC087491.1, MEG3, AC019077.1, AC073592.1, LINC01913, AC116407.1, AC005562.1, AL161756.1, AC010168.1, AC112512.1, POLR2J4, AP002358.1, AC090921.1, LINC00612, AC004540.1, AL772337.1, LINC00243, LINC01588, AC104472.1, HLA-F-AS1, AP000317.1, AC005323.1, AC129492.1, AL645728.1, AL008729.1, AC131097.1, CD27-AS1, ALOX12-AS1, AC025171.2, LINC00588, AC025262.1, LINC00269, AC026790.1, LINC01020, LINC02449, AL137145.2, AC116351.1, DHRS4-AS1, AC135983.1, FAM66B, MIR99AHG, LL22NC01-81G9.3, MIR17HG, MCM3AP-AS1, BX322557.1, ZNF663P, AATBC, BCRP3, LINC00598, FAM230B, LINC00189, AC114730.1, ARHGAP27P1-BPTFP1-KPNA2P3, LINC01139, AC239803.1, LINC01356, LY86-AS1, AC009403.1, AC007249.1, AC073316.1, AC073263.1, AL109955.1, PP14571, LINC00339, MIF-AS1, AC008060.1, AC011298.1, AC125494.1, AC019155.2, AC008770.1, AL445238.1, LINC02076, AC093802.1, Z85994.1, LL22NC03-63E9.3, AL589947.1, IGBP1-AS2, PPP3CB-AS1, GAS8-AS1, C7orf65, LINC01465, C1orf229, EXOC3-AS1, AC092675.1, AC106876.1, C7orf71, LINC01118, AC064874.1, AC005481.1, AC011997.1, AC062017.1, AC112721.1, LINC01793, AC023469.1, AC112721.2, LINC01124, AC079354.1, CYTOR, AC079305.1, AL031587.1, AC137630.1, AL590666.1, AC005104.1, LINC01778, LINC02068, AL445685.1, CLDN10-AS1, AL118511.1, AC096677.1, MEG9, AL009050.1, LINC00473, TH2LCRR, LMO7DN-IT1, AC004471.1, AL353801.1, LINC01825, AL117338.1, AL441992.1, NUTM2A-AS1, LINC01615, AC092198.1, AL391097.1, AL731537.1, AL646090.1, AL683807.1, AC093690.1, AC092598.1, RABGAP1L-IT1, AL359094.1, HLA-DQB1-AS1, AL109947.1, AL024497.1, LINC00630, AC098613.1, AC005165.1, AP001599.1, DHRSX-IT1, AL355001.1, AL138733.1, DSCR4-IT1, LINC01044, LINC01120, AL591624.1, AL121990.1, AC008277.1, AC002463.1, AC106786.1, AL078459.1, AC245052.1, AC093117.1, LINC00571, DIP2A-IT1, FO393418.1, AF230666.1, RAET1E-AS1, LINC01422, AC069213.1, AL109797.1, LINC01208, AC009226.1, AC034195.1, AC098828.2, AC112495.1, CCDC18-AS1, MIR503HG, AC116609.1, AC008073.1, AL645608.1, LINC00205, AC006145.1, AL513217.1, LGALS8-AS1, LINC01983, LINP1, AL357507.1, UBE2E1-AS1, AL669970.1, ENTPD3-AS1, IL10RB-AS1, LINC00114, AL589684.1, AC125608.1, AC007255.1, DIAPH3-AS2, AL603910.1, LINC01342, LINC01870, AC004870.2, AC005006.1, AL161935.1, AL645941.1, AC007091.1, FAM95B1, AL360093.1, EFCAB6-AS1, MYCNUT, HRAT92, LINC01078, ABCC5-AS1, LINC01707, AC068481.1, OSER1-AS1, AP001469.1, AC010136.1, MIR4432HG, AC109779.1, AC008074.1, AL513008.1, AC016738.1, ROR1-AS1, LINC01767, AC009948.1, AC002456.1, SMCR2, AC104809.1, LINC01441, LINC01449, MIR181A2HG, FLJ37035, AL450344.1, TCEAL3-AS1, EPB41L4A-AS1, AL355303.1, CCNT2-AS1, AC005076.1, AC108681.1, AL008723.1, EGFR-AS1, AC007319.1, AL049795.1, LINC00691, SNHG14, AC091729.1, AC245452.1, AL109613.1, AC104823.1, AP001630.1, ELMO1-AS1, INHBA-AS1, POU6F2-AS1, AC099786.1, AC112715.1, LINC01857, AC000123.1, MIR548XHG, AMMECR1-IT1, AC099786.2, AC009506.1, LINC02054, HCG14, AL133264.2, DNAJC27-AS1, AL390729.1, AL137802.1, LINC00570, MIR3681HG, C5orf66, HAGLR, AC008278.1, AC022400.1, PHEX-AS1, LINC00466, AL606469.1, AC234781.1, AC107072.1, AC104699.1, VSTM2A-OT1, AL031599.1, WARS2-IT1, AC090044.1, AL391427.1, LINC01133, AL023754.1, AP000697.1, AL117329.1, AC131097.3, AL355388.1, SLC25A5-AS1, LINC01142, AF196972.1, AL161785.1, LINC01527, LINC01567, AL590640.1, CHL1-AS2, AC007879.1, AL365226.1, AL356966.1, AL451074.2, AC011239.1, FP700111.1, AL391361.2, AC009276.1, AC017104.1, LINC00703, LINC01280, GPC6-AS2, SMIM25, LINC02052, AC114489.1, AP001476.1, AL606970.1, STK24-AS1, PRKAR2A-AS1, LINC00539, LINC01708, AL450998.2, AL354953.1, AL356417.1, AC245100.1, TTC21B-AS1, AC025428.2, AC138150.1, AL078594.1, AC010884.1, LINC00365, AC109309.1, AL591686.1, TMLHE-AS1, AC096677.2, AP001439.1, AC017067.1, LINC01087, LINC01754, LINC01886, COL18A1-AS2, LINC01117, AL356277.2, AL139158.2, ZMIZ1-AS1, SVIL-AS1, AL080284.1, HSD52, AC095032.1, TNK2-AS1, RTCA-AS1, AL451042.1, AL391095.1, AC106900.1, AC064871.1, AL135787.1, AF124730.1, LINC00885, LINC01823, AL513164.1, SH3BP5-AS1, Z84484.1, AC009227.1, RASAL2-AS1, AL133553.1, AL390038.1, LAMTOR5-AS1, E2F3-IT1, AC025165.1, AC024600.1, Z82186.1, AC098936.1, AC007182.1, PCOLCE-AS1, AC016716.2, AL034349.1, AC099850.1, AC016735.1, TEX26-AS1, AC063965.1, AC015987.1, AL391704.1, AL353768.1, AP000704.1, AL022326.1, NTM-AS1, AL139397.1, LINC00853, AL355482.1, TMEM72-AS1, AC010789.1, COL4A2-AS2, THRB-IT1, RORB-AS1, LINC01689, AL161908.1, LINC00240, AC107079.1, AL133351.1, AL589843.1, LINC00393, CDKN2A-AS1, OPA1-AS1, LINC01691, LINC01398, AC009518.1, AL391244.1, AC083949.1, TSSC1-IT1, AC138028.2, AL160162.1, POT1-AS1, AC019155.3, AC005534.1, AP001347.1, LINC01766, LINC00863, LINC00320, LINC02262, AL391684.1, LINC00184, AL663023.1, AL353150.1, AL390066.1, LINC01266, PGM5-AS1, AC017002.1, LINC01752, KCNC4-AS1, LINC01645, AL645608.2, AL513412.1, LARGE-AS1, INE1, AL121983.1, AL590714.1, AL445645.1, LINC01526, AC010082.1, AL732323.1, AC000067.1, AC096541.1, AL355483.1, AL162586.1, EIF1AX-AS1, LINC01058, AL121894.1, AC012485.1, CATIP-AS1, AC093422.2, AL603832.1, WWC3-AS1, LINC00337, GRTP1-AS1, AL450226.1, AL583808.1, AL355336.1, AL021368.1, AL162457.2, AC092484.1, AC083900.1, AL157392.1, LINC00237, LINC00972, AL035250.1, SLC9A3-AS1, AL358216.1, AC018643.1, AC073957.1, AL160290.1, AC012354.1, LINC00618, AC012462.1, AC096631.1, AL662890.2, OSTM1-AS1, AL590617.2, AL121584.1, AC073283.1, LINC00092, AC078962.1, AC078883.1, MIR137HG, AC092637.1, AC073367.1, AC079790.1, AC007362.1, AP001628.1, LINC01795, AC008937.1, LINC02470, TRAPPC12-AS1, INTS6L-AS1, AL021026.1, LINC01297, PCDH9-AS3, TAF1A-AS1, LINC00705, AL158013.1, AC018693.1, LINC01770, AL158212.1, LINC00113, AL139240.1, AL591623.1, AC023283.1, AL024474.2, AL513327.1, AL591178.1, AL583804.1, LINC01594, LHFPL3-AS2, LINC01678, AL391811.1, AC016027.1, AL354740.1, AC079630.1, PPP1R26-AS1, AC078942.1, NRSN2-AS1, SFTA1P, AL591896.1, NHEG1, AC121247.1, AC025188.1, AL136980.1, CR786580.1, AP000345.2, AC104134.1, AC019330.1, LINC01504, BOLA3-AS1, MPRIP-AS1, LINC01763, AL021707.1, AL049812.2, RFPL1S, JPX, AL136366.1, NUTM2B-AS1, AL354707.1, LINC01107, AC002064.1, CYP4A22-AS1, LINC00475, LINC01703, AL365434.1, TTTY16, AC005237.1, AL450384.1, LINC01393, LINC01821, AC002480.1, ZNF385D-AS1, AL162399.1, LINC02476, LINC01980, AP000320.1, LINC01492, DOCK4-AS1, NCBP2-AS1, EDNRB-AS1, AC096669.1, LINC01623, MTOR-AS1, AC005281.1, LINC02158, LINCMD1, MYT1L-AS1, AGBL4-IT1, C9orf135-AS1, AL161644.1, AP001046.1, LINC01729, AL606491.1, AC005487.1, AC064875.1, KCNMA1-AS3, BX255923.1, LINC01716, CADM3-AS1, LINC01771, PACRG-AS3, FAM225B, AC114737.1, PTPRD-AS1, AL354984.2, AC092100.1, AL592166.1, FAM66E, AL096772.1, AP001627.1, FGD5-AS1, LINC02036, AC017002.2, MEG8, AL357500.1, AC087501.1, DBH-AS1, AL596247.1, LINC01389, P3H2-AS1, DHRS4L1, AL049637.1, AL137220.1, PROSER2-AS1, MIAT, AL109610.1, TRAM2-AS1, AC004540.2, AL080250.1, AC073321.1, AC025918.1, AL121917.1, AC069281.1, UBXN7-AS1, LINC00626, TRMT2B-AS1, AL355490.1, RUSC1-AS1, AL162431.1, LINC02072, AL022315.1, LINC00368, PSG8-AS1, AL109924.1, LINC00115, LINC01456, AC098872.1, AC023590.1, AL445490.1, AC012368.1, AL513365.2, AL035404.2, AL391244.2, AL138767.3, AL671511.1, AC000036.1, LINC02249, PCA3, AL109741.1, AL121574.1, ODF2-AS1, AL512444.1, SATB2-AS1, AL050320.1, AC009950.1, NRIR, ABHD11-AS1, AL500522.1, AC005550.1, LINC01534, HAR1A, AC102953.1, AL355482.2, UBXN10-AS1, LAMP5-AS1, AL591468.1, AL513303.1, BX005266.2, KCNIP2-AS1, AP001434.1, PRICKLE2-AS3, AC092802.1, LINC01772, FGF13-AS1, AP000561.1, ZNF503-AS1, LINC01776, AC009531.1, LINC00623, SLC39A12-AS1, AC073283.2, AL158839.1, AL122017.1, LINC00937, AC099342.1, LINC02097, AP001476.2, LINC01907, BAIAP2-AS1, AL356124.1, AC124944.1, AL513185.1, AP4B1-AS1, AL139420.1, LINC00685, AC010536.1, AL009176.1, AL583785.1, SGMS1-AS1, LINC01733, LEMD1-AS1, GAS1RR, AL031658.1, LINC00381, ZNF32-AS1, AL365271.1, AL451060.1, AL135960.1, GRM7-AS3, ISM1-AS1, AC009961.1, ARHGAP26-AS1, AC093382.1, AL031123.1, AC091729.2, AL122058.1, AL022157.1, CFLAR-AS1, LINC00351, LINC01811, NUP50-AS1, AL606489.1, AL354836.1, AL359182.1, AC099066.1, PSPC1-AS2, AL353803.1, HAGLROS, ST7-AS2, LINC00375, AL022310.1, AC084809.1, AC016831.1, LINC01876, PARD3-AS1, C12orf77, AC013480.1, AL450332.1, MRPL23-AS1, SLC16A1-AS1, AC093642.1, AC135371.1, AL442071.1, AL365214.2, BX322234.1, AL355294.1, AL121787.1, AL390198.1, Z93930.2, LINC01748, LINC01035, LINC00323, AL593854.1, LINC01918, UPK1A-AS1, KIRREL-IT1, AC004994.1, AL049569.1, AP000289.1, AL158055.1, BTBD9-AS1, AC016722.1, CYP4F26P, LINC00606, AL158850.1, AL132657.1, AC092634.3, AL450344.2, AL359979.1, PAPPA-AS2, AL390067.1, LINC00343, AC092155.1, SHANK2-AS1, LINC00974, AL357793.1, AL513314.1, AL121899.1, AP006216.1, AL365356.1, PLCG1-AS1, AC019118.1, AC021028.1, AC004973.1, AL354977.1, LINC01108, TEX41, AL606748.1, LINC01535, ENTPD1-AS1, LENG8-AS1, AL360181.1, MIR217HG, AL161452.1, FGF12-AS3, FAM66C, LINC01709, U62317.1, AL020994.2, SMCR5, AC007966.1, AF127936.1, AL606760.1, AC007365.1, PP12613, DISC1-IT1, DAB1-AS1, AL451164.1, AC000124.1, FAM30A, NAALADL2-AS2, AC117465.1, AC109826.1, AC005828.1, AL138749.1, CACTIN-AS1, AL136311.1, AC011893.1, LINC00840, AL117382.1, AC005082.1, MEIS1-AS3, AL390036.1, AC006001.2, LINC01509, AL591885.1, AC092164.1, AC097059.1, AL158834.1, LINC00348, AL109811.1, AC004112.1, AC010894.2, AL451105.2, AC093901.1, AC104463.2, LHFPL3-AS1, AC135178.1, AC002472.1, AC092801.1, AL354733.1, AL035416.1, AL359541.1, Z97652.1, LINC01359, AL731577.1, AL451069.1, BSN-AS2, LINC01276, LINC00454, AC007271.1, LINC00161, AC062021.1, AL031847.1, DANCR, AC010890.1, AL023802.1, AL358075.1, AC078883.2, AC092167.1, AL391845.1, MAGI2-AS2, LINC01692, LINC01203, AC025946.1, AC012593.1, LINC00658, AP000477.1, AL136988.2, LINC01247, C17orf112, AL158035.1, AC007285.1, AC007036.1, SLC8A1-AS1, LINC00511, ITGB2-AS1, AL589765.1, AC105446.1, LINC00629, Z98257.1, AC104170.1, FOCAD-AS1, AL158166.1, LINC02094, AC244021.1, L29074.1, AL031767.1, AL512785.1, AC025822.1, AC104667.1, LMCD1-AS1, LINC01630, HORMAD2-AS1, LBX1-AS1, GCSAML-AS1, AC241644.1, LINC01153, IL21-AS1, AL669970.2, AL161725.1, AC092755.1, WDR11-AS1, AF178030.1, AC011290.1, AC018511.1, MGAT3-AS1, AC092535.1, AL023581.2, AC244453.1, MIR663AHG, AC009365.2, ST7-AS1, AL162724.1, AC079145.1, SPATA13-AS1, HCG15, AL355613.1, AL157935.1, AL133346.1, AC017101.1, AL606534.1, AL672291.1, AL356272.1, FAM155A-IT1, AC105760.2, AL158834.2, MIS18A-AS1, SMIM2-AS1, LINC01985, AL603839.1, AC110015.1, LINC01364, AC009229.1, AL035541.1, AC010998.1, AC009502.1, RBM26-AS1, AL359644.1, AC017074.1, AL121983.2, AL157832.1, DLG1-AS1, AL133410.1, HPN-AS1, KIF9-AS1, LINC01806, AC245884.1, ZMYM4-AS1, PCGEM1, CSE1L-AS1, AC053503.1, AP001471.1, AL450345.1, LINC00310, AL390838.1, LINC01537, STK4-AS1, AC124861.1, AL157702.2, CR559946.1, AL445472.1, NCAM1-AS1, AL138921.1, AC004771.1, AC099066.2, LINC01268, LINC01624, AL592301.1, AL160163.1, LINC01483, AL928970.1, AC096540.1, SLC2A1-AS1, AL357315.1, DNAJC9-AS1, AC092614.1, SPAG5-AS1, AC018647.1, AL590422.1, LINC00376, AL122019.1, CNTN4-AS2, AL136528.1, AL031316.1, Z94721.1, AL031676.1, AL031779.1, AL583839.1, AC005392.1, CERS6-AS1, AL391056.1, AL080276.2, LINC01132, LINC01714, SOX21-AS1, STEAP2-AS1, CLYBL-AS2, UST-AS1, AL162734.1, LINC00355, LINC01068, AL355581.1, AL033504.1, AL358394.1, LINC02088, DNMBP-AS1, AP001619.1, AL354892.1, AL391097.2, AL713998.1, AC092159.1, AP000459.1, AC006042.1, AC239809.3, AL513329.1, AL121987.2, LINC01940, AL138916.1, LINC01693, AC072062.1, ASH1L-IT1, AC002553.1, AL158198.1, FAM212B-AS1, AL590408.1, SUCLA2-AS1, AL358075.2, AC073486.1, AL590652.1, FAM66A, AL731569.1, SNAP25-AS1, FLJ31104, LINC02344, AL136979.1, LINC01655, AL357874.1, AL590006.1, AC007383.2, LINC01738, LINC01341, TARID, AL451042.2, AL355488.1, LINC01429, AC105935.1, AC073343.2, AL162591.1, RAPGEF4-AS1, AL158835.1, HCG20, AC069257.1, AC078842.1, AC010967.1, AL512638.2, AL139246.3, AC114763.1, AL160408.1, LINC01736, AL365277.2, AC131571.1, LYPLAL1-AS1, AC023469.2, LINC01515, AC133106.1, AC012368.2, AC118553.1, AL589990.1, LINC01820, AL392172.1, AP000692.1, AC092839.1, MELTF-AS1, AC003991.1, AL161725.2, AL096678.1, FALEC, LINC01649, AC099684.1, LINC01494, AP001469.2, LINC01402, AL031283.1, AL354861.2, AL357140.2, AC004941.1, AC007952.2, AL020996.1, AL162385.1, AL592486.1, AL512353.1, AL022341.1, RNF144A-AS1, AC004830.1, C1orf143, AL954650.1, AL355607.1, LINC00578, AC073050.1, HCG11, AP001476.3, EFCAB14-AS1, AC093495.1, AC012442.1, AC096639.1, AL162742.1, LINC01320, RALY-AS1, LINC02048, AL021707.2, AL731568.1, PCAT6, TBX18-AS1, AL049712.1, AL512770.1, LINC01209, LINC01350, GUSBP11, AL158070.1, AP001610.1, AC008440.1, MIR646HG, AC115618.2, CELSR3-AS1, AL354989.1, LINC02263, AC015971.1, AC106873.1, TXNDC12-AS1, ITPKB-IT1, AC007040.1, AL031668.1, AL162391.1, LINC01004, AL356481.1, LINC01635, HSPC324, AP001468.1, AL031056.2, AL022068.1, LINC01185, AL360182.2, LINC01768, LINC00687, AL357552.2, AL590764.1, AC004951.1, AL139260.1, LINC02474, AL137244.1, AC098484.1, LINC01546, AP006222.1, AL929236.1, U51244.1, AL137025.1, AL450263.1, LINC01013, AC106875.1, AC011897.1, AL513550.1, AC006460.1, AL160272.1, MIR34AHG, AC016396.1 |

,
