# Supplementary material for: Identification of Immune-Related Prognostic Genes and LncRNAs Biomarkers Associated With Osteosarcoma Microenvironment
Source: Front Oncol. 2020 Jul 24;10:1109. doi: 10.3389/fonc.2020.01109 (PMC7393189; doi:10.3389/fonc.2020.01109)
Supplement: Supplementary Table 2 — List of Immune-related lncRNAs. [file Table_2.DOCX]

| **Module** | **Genes** |
| --- | --- |
| Red (n=62) | PML, SOD1, IFNAR1, IFNAR2, IFIH1, PSMD4, MX2, STAT1, TAP2, OAS1, AKT1, IL10RB, IRF1, APOBEC3F, TRIM22, EIF2AK2, HLA-A, BST2, MX1, DDX58, ADAR, PSMB8, DHX58, TLR3, ISG15, HLA-C, FPR2, APOBEC3G, ADIPOQ, IFNGR2, RFX5, TAP1, OASL, HLA-B, APOBEC3A, IFITM1, RSAD2, HLA-E, B2M, PLSCR1, FLT3LG, CCL8, IFNB1, JAK2, IL15RA, CCL2, ACTA1, ISG20, SECTM1, AQP9, TNFSF10, HLA-F, KCNH2, KIR2DL4, IL12A, CCL7, CNTFR, IRF7, IL22RA1, IL27, IDO1, TNFSF18 |
| Brown (n=180) | PSMC2, ANXA6, DAXX, ACTG1, AP3B1, SLC29A3, LMBR1, RAC1, MC1R, IL6ST, THRA, TOR2A, MAPK14, PGRMC2, TRIM5, GRB2, MICA, TBK1, PPP4C, IL13RA1, CMTM3, GBP2, NFYB, PLXNA2, GMFB, KLRC3, CSRP1, PSMC6, MAVS, NR3C1, TMSB10, IL7, NOD1, RXRA, S100A11, PSMD3, BCL10, NUDT6, SKIV2L, BPHL, ILK, CAT, LRSAM1, CD40, CNTF, GDF9, OPRL1, NR6A1, F2R, PPARD, PPP3CB, CMTM6, CSF1, PIK3CG, CD81, BECN1, JAK1, PLXNB2, TAPBPL, FAM3B, NLRX1, BRD8, NFYC, TRPC4AP, TGFBR2, PSMD7, IL17RC, TRIM27, HSPA8, RXRB, NFATC3, KLKB1, TANK, PIK3CA, TK2, ZC3HAV1, TMEM173, PF4, CSF1R, MAPK3, VDR, SLIT2, CD4, RABEP2, RAC2, NENF, LAT, SRC, TLR2, FAM19A5, TNFRSF11A, IFI30, PSMD14, ARRB1, NR2C1, CCRL2, BRAF, NFATC1, ISG20L2, GRN, FCER1G, DDX17, TNFRSF10A, CRLF3, NR1H2, NRAS, ANGPTL6, IRF3, GNRH2, CCR1, SHC1, CKLF, PTK2B, CBLB, LTB4R, NFKBIE, LECT2, CD72, GDF7, LMBR1L, ADIPOR1, TNFRSF25, TEC, TGFB2, AMH, SDC2, IRF5, CSH1, SHC3, TNFRSF14, AGER, FGF18, GCGR, EPO, MMP9, IL17RA, SEMA7A, SOCS1, THPO, PDGFB, RELB, SEMA4C, GHRL, FAM3D, LTB4R2, TNFSF15, NFKBIA, RNASE3, NOD2, S100A3, MLN, PLXNA1, BMP10, MTNR1A, HNF4G, HTR3E, NRG2, GNRH1, PTX3, SEMA4D, NFKBIZ, IL18RAP, UNC93B1, RXFP2, CHP2, INSL6, TMPRSS6, IKBKB, BMP7, LTBP3, TNFSF11, DEFB1, PDF, UCN3, SEMA5A, SEMA6B, SPP1, CSH2, PI3, NODAL |
